# Hyperoxia for accidental hypothermia and increased mortality: a post-hoc analysis of a multicenter prospective observational study

**DOI:** 10.1186/s13054-023-04407-8

**Published:** 2023-04-01

**Authors:** Ryo Yamamoto, Jo Yoshizawa, Shuhei Takauji, Mineji Hayakawa, Junichi Sasaki

**Affiliations:** 1 Department of Emergency and Critical Care Medicine, Keio University School of Medicine, 35 Shinanomachi, Shinjuku, Tokyo 160-8582 Japan; 2 Department of Emergency Medicine, Asahikawa Medical University Hospital, Asahikawa, Japan; 3 Department of Emergency Medicine, Hokkaido University Hospital, Sapporo, Japan

**Keywords:** Hyperoxemia, Arterial partial pressure of oxygen, Oxygen toxicity, Reactive oxygen species, Severe hypothermia

## Abstract

**Background:**

Supraphysiologic oxygen administration causes unfavorable clinical outcomes in various diseases, including traumatic brain injury, post–cardiac arrest syndrome, and acute lung injury. Accidental hypothermia is a critical illness that reduces oxygen demands, and excessive oxygen is likely to emerge. This study aimed to determine whether hyperoxia would be associated with increased mortality in patients with accidental hypothermia.

**Methods:**

A post-hoc analysis of a nationwide multicenter prospective observational study (ICE-CRASH study) on patients with accidental hypothermia admitted in 2019–2022 was conducted. Adult patients without cardiac arrest whose core body temperature was < 32 °C and whose arterial partial pressure of oxygen (PaO_2_) was measured at the emergency department were included. Hyperoxia was defined as a PaO_2_ level of 300 mmHg or higher, and 28-day mortality was compared between patients with and without hyperoxia before rewarming. Inverse probability weighting (IPW) analyses with propensity scores were performed to adjust patient demographics, comorbidities, etiology and severity of hypothermia, hemodynamic status and laboratories on arrival, and institution characteristics. Subgroup analyses were conducted according to age, chronic cardiopulmonary diseases, hemodynamic instability, and severity of hypothermia.

**Results:**

Of the 338 patients who were eligible for the study, 65 had hyperoxia before rewarming. Patients with hyperoxia had a higher 28-day mortality rate than those without (25 (39.1%) vs. 51 (19.5%); odds ratio (OR) 2.65 (95% confidence interval 1.47–4.78); *p* < 0.001). IPW analyses with propensity scores revealed similar results (adjusted OR 1.65 (1.14–2.38); *p* = 0.008). Subgroup analyses showed that hyperoxia was harmful in the elderly and those with cardiopulmonary diseases and severe hypothermia below 28 °C, whereas hyperoxia exposure had no effect on mortality in patients with hemodynamic instability on hospital arrival.

**Conclusions:**

Hyperoxia with PaO_2_ levels of 300 mmHg or higher before initiating rewarming was associated with increased 28-day mortality in patients with accidental hypothermia. The amount of oxygen to administer to patients with accidental hypothermia should be carefully determined.

*Trial Registration*: The ICE-CRASH study was registered at the University Hospital Medical Information Network Clinical Trial Registry on April 1, 2019 (UMIN-CTR ID, UMIN000036132).

**Supplementary Information:**

The online version contains supplementary material available at 10.1186/s13054-023-04407-8.

## Background

Oxygen administration has been a vital treatment for critically or acutely ill patients [1, 2]. However, supraphysiological levels of oxygen in the blood and/or tissue have been linked to unfavorable clinical outcomes in a variety of diseases, including traumatic brain injury, severe/multiple injuries, post–cardiac arrest syndrome, and post–cardiac surgery [3–6]. In addition, inappropriately high fraction of inspired oxygen (FiO_2_) was associated with increased mortality in critically ill patients with conditions such as sepsis and respiratory failure [7, 8].

While the pathophysiological mechanisms underlying the harmful effects of hyperoxia have been investigated, oxidative toxicity in the brain and pulmonary tissues has been identified as a key factor influencing clinical outcomes in critically ill patients [9, 10]. Some studies showed that hyperoxia causes cerebral vasoconstriction and mitochondrial dysfunction in the damaged brain, which paradoxically reduces oxygen delivery to the cerebral tissues [10, 11]. Furthermore, unnecessary reactive oxygen species were detected in patients who experienced hyperoxia during mechanical ventilation, resulting in pulmonary vasoconstriction and alveolar injuries [9, 12]. Hyperoxia-induced acute lung injury (ALI) was another adverse event caused by redundant oxygen, and synergistic tissue toxicity caused by systemic inflammation and hyperoxia has been suggested [12].

Accidental hypothermia is a critical illness that necessitates a variety of resuscitative measures, particularly when the core temperature falls below 32 °C [13, 14]. While optimal rewarming methods have been investigated [14, 15], the literature on appropriate tissue oxygen tension until temperature recovery is limited. Given that hypothermia is known to reduce oxygen demand in several organs [16], excessive oxygen molecules that cannot be utilized by the tissue are likely to emerge. Therefore, the unfavorable effects of hyperoxia would manifest in patients with hypothermia, even though the amount of oxygen should be appropriately titrated to meet the increasing demand of the tissues while rewarming.

Accordingly, this study conducted a post-hoc analysis on a multicenter prospective observational study on accidental hypothermia. The study aimed to determine whether hyperoxia would be associated with unfavorable clinical outcomes in patients with accidental hypothermia, with the hypothesis that hyperoxia prior to the initiation of in-hospital rewarming was associated with increased 28-day mortality after hospital arrival.

## Methods

### Study design and setting

This was a post-hoc analysis of a nationwide multicenter prospective observational study that was conducted by the Intensive Care with Extra Corporeal membrane oxygenation Rewarming in Accidentally Severe Hypothermia (ICE-CRASH) study group from December 2019 to March 2022 [17, 18]. The ICE-CRASH study included patients with accidental hypothermia at participating 36 tertiary care centers and was registered at the University Hospital Medical Information Network Clinical Trial Registry on April 1, 2019 (UMIN-CTR ID, UMIN000036132) prior to study initiation. The ICE-CRASH study was supported by the Japanese Association for Acute Medicine (approval no. 0005) and approved by the institutional review board for conducting research with human participants at the head institute of the ICE-CRASH study group (approval no. 18194 from Asahikawa Medical University). This study was conducted in accordance with the Helsinki Declaration, and written informed consent was waived due to the anonymity of the data.

The ICE-CRASH study enrolled consecutive patients aged 18 years or older with accidental hypothermia, including those with cardiac arrest, and hypothermia was defined as a core body temperature less than 32 °C measured at the emergency department (ED) on arrival. As there was no validated uniform rewarming strategy for accidental hypothermia during the study period, rewarming procedures were decided by an attending physician based on patient conditions such as hypothermia severity, vital signs, and the presence of cardiac arrest. Rewarming methods included blankets, warm parenteral fluids, warm baths, body cavity lavage, intravascular thermoregulated catheters, hemodialysis, and extracorporeal membrane oxygenation. Rewarming was typically initiated in the ED under cardiopulmonary monitoring and continued after intensive care unit (ICU) admission.

### Study population

Data from the ICE-CRASH study (2019–2022) were reviewed retrospectively. Patients with accidental hypothermia were included if they were (1) 18 years old or older, (2) diagnosed with a body temperature less than 32 °C, and (3) had available arterial partial pressure of oxygen (PaO_2_) data obtained at the ED. Patients who were in cardiac arrest when they arrived at the hospital were excluded because previous studies have suggested that hyperoxia can be harmful in post–cardiac arrest syndrome.

### Data collection and definition

Patient data for the ICE-CRASH study were prospectively collected and entered into an online data collection portal at each hospital. Age, sex, Charlson comorbidity index, the activity of daily living (ADL), the etiology of hypothermia, the place of occurrence of hypothermia, transportation time from the scene to the hospital, vital signs on hospital arrival, presence of cardiac arrest on hospital arrival, rewarming methods, laboratory investigations and arterial blood gas assay that was obtained at the ED on arrival and at the ED or ICU after rewarming to 36 °C and was corrected by body temperature as appropriate at each institution, time from arrival to rewarming to 33 °C and 36 °C, Sequential Organ Failure Assessment (SOFA) score on admission, and mortality on the day of admission and during rewarming were all recorded. In addition, length of ICU and hospital stay, duration of ventilator use, cerebral performance category (CPC) at discharge, survival status 28 days after admission, and adverse events related to hypothermia/rewarming (ventricular fibrillation, hemorrhage, pneumonia, pancreatitis, and acute kidney injury) were all available.

According to previous research on hyperoxia in other diseases, hyperoxemia was defined as a PaO_2_ level of 300 mmHg or higher [2, 4, 19]. Hyperoxia before the initiation of in-hospital rewarming was defined as hyperoxia at the ED on arrival. Trajectory of hyperoxia during rewarming was defined as average change in PaO_2_ per hour until rewarming to 36 °C, that was calculated using PaO_2_ before and after rewarming and the time duration of rewarming. Severe hypothermia was defined as a core body temperature of less than 28 °C [13, 20], and hemodynamic instability was defined as a systolic blood pressure (SBP) of less than 90 mmHg. The database lacked detailed indications for each rewarming procedure as well as hemodynamic status before, during, and after rewarming.

### Outcome measures

The primary outcome was 28-day mortality. Secondary outcomes included favorable neurological function at discharge (defined as a CPC of 2 or lower), ICU-, hospital-, and ventilator-free days to 28 days after admission, and the frequency of adverse events related to hypothermia/rewarming.

### Statistical analysis

The primary outcome was compared between patients with and without hyperoxia using the Chi-square test as an unadjusted analysis. Then, inverse probability weighting (IPW) using propensity scores was conducted to adjust background characteristics between patients with and without hyperoxia [21, 22]. The propensity score for weighting was developed using a logistic regression model fitted with generalized estimating equations (GEE) to estimate the probability of hyperoxia exposure and account for within‐institution clustering [23]. Based on previous studies, relevant covariates were carefully selected from known or potential predictors for receiving supraphysiologic amounts of oxygen and predicting clinical outcomes in patients with accidental hypothermia (Additional file 1: Figure S1) [14, 20, 24–26]. These covariates included age, sex, Charlson comorbidity index, ADL (independent/with limited help vs. considerably/completely dependent), etiology of hypothermia (intoxication, infection, and trauma), place of occurrence of hypothermia (indoor vs. outdoor), transportation time, vital signs on hospital arrival (Glasgow Coma Scale (GCS), SBP, heart rate, and respiratory rate), hypothermia severity, and arterial blood gas assay (lactate and base excess). Laboratory data on arrival, such as hematocrit, platelet count, prothrombin time, creatinine level, and glucose level, were also included as covariates because they are considered survival predictors in accidental hypothermia and are unaffected by PaO_2_ on arrival [21, 26]. On the other hand, covariates related to rewarming information were not included in the model because hyperoxia exposure would affect such covariates [26]. Patients with missing covariates were excluded from the propensity score calculation. The discrimination ability of the propensity score was analyzed using the *c*-statistic [22]. The weight was calculated as the inverse of the propensity score of hyperoxia exposure. To avoid extreme weight based on propensity scores, patients with a propensity score of 0.05 or less or 0.95 or higher were excluded from the IPW analyses. The primary outcome was compared using the Chi-square test, and secondary outcomes were compared using odds ratios (ORs) or the Mann–Whitney *U* test [22].

Three sensitivity analyses were performed in order to validate the primary results. First, to validate results that were not dependent on the propensity score calculation, generalized estimating equation analysis with the logit link function was used to adjust patient backgrounds and differences in quality of care between participating hospitals [23]. Second, multivariate logistic regression was conducted with covariates for the propensity score calculation to confirm that the results were not dependent on the propensity score or within-institution clustering. Third, IPW was conducted with no restriction on the propensity score to validate that extreme weight truncation was appropriate [21, 22].

In addition, restricted cubic spline curves for estimating 28-day mortality by PaO_2_ at the ED were created to identify any PaO_2_ thresholds that affect the clinical outcomes of accidental hypothermia. For this, a generalized additive model was adopted. Then, to explore the ranges of hyperoxia that would affect outcomes, two different cut-offs were chosen from the spline curves and the hyperoxia was re-defined, with which the IPW analyses were repeated.

Moreover, as the effect of trajectory of hyperoxia would be expected to affect clinical outcomes, it was entered into a post-weight logistic regression model along with hyperoxia. In addition, another post-weight logistic model was analyzed, in which rewarming methods, time duration of rewarming, and the trajectory of hyperoxia were entered with hyperoxia.

Subgroup analyses were performed to investigate the relationships between hyperoxia, clinical characteristics, and 28-day mortality. Targeted subgroups were selected based on previous research into the clinical outcomes of patients with accidental hypothermia. The IPW analyses of the primary outcome were repeated in patient subgroups divided by age (< 65 vs. ≥ 65 years), presence of chronic cardiopulmonary diseases such as congestive heart failure and chronic lung disease, hemodynamic instability on hospital arrival (SBP ≥ 90 vs. < 90 mm Hg), and hypothermia severity (core body temperature < 28 °C vs. ≥ 28 °C). Subgroup analyses were also conducted in patients without hypoxia, defined as a PaO_2_ level less than 60 mmHg.

Descriptive statistics are presented as a median (interquartile range (IQR)) or a number (percentage). The results were presented as a standardized difference and a 95% confidence interval (CI). The balance of covariates before and after weighting was evaluated with a standardized difference, in which less than 0.1 was considered insignificant [21]. The hypothesis was tested on the primary and secondary outcomes, with a two-sided *α* threshold of 0.05 considered significant. All statistical analyses were conducted using the IBM Statistical Package for the Social Sciences Statistics for Windows Version 28.0 (IBM Corp., Armonk, NY) and R Version 4.0.2 (R Foundation for Statistical Computing, Vienna, Austria).

## Results

### Patient characteristics

**Fig. 1:**
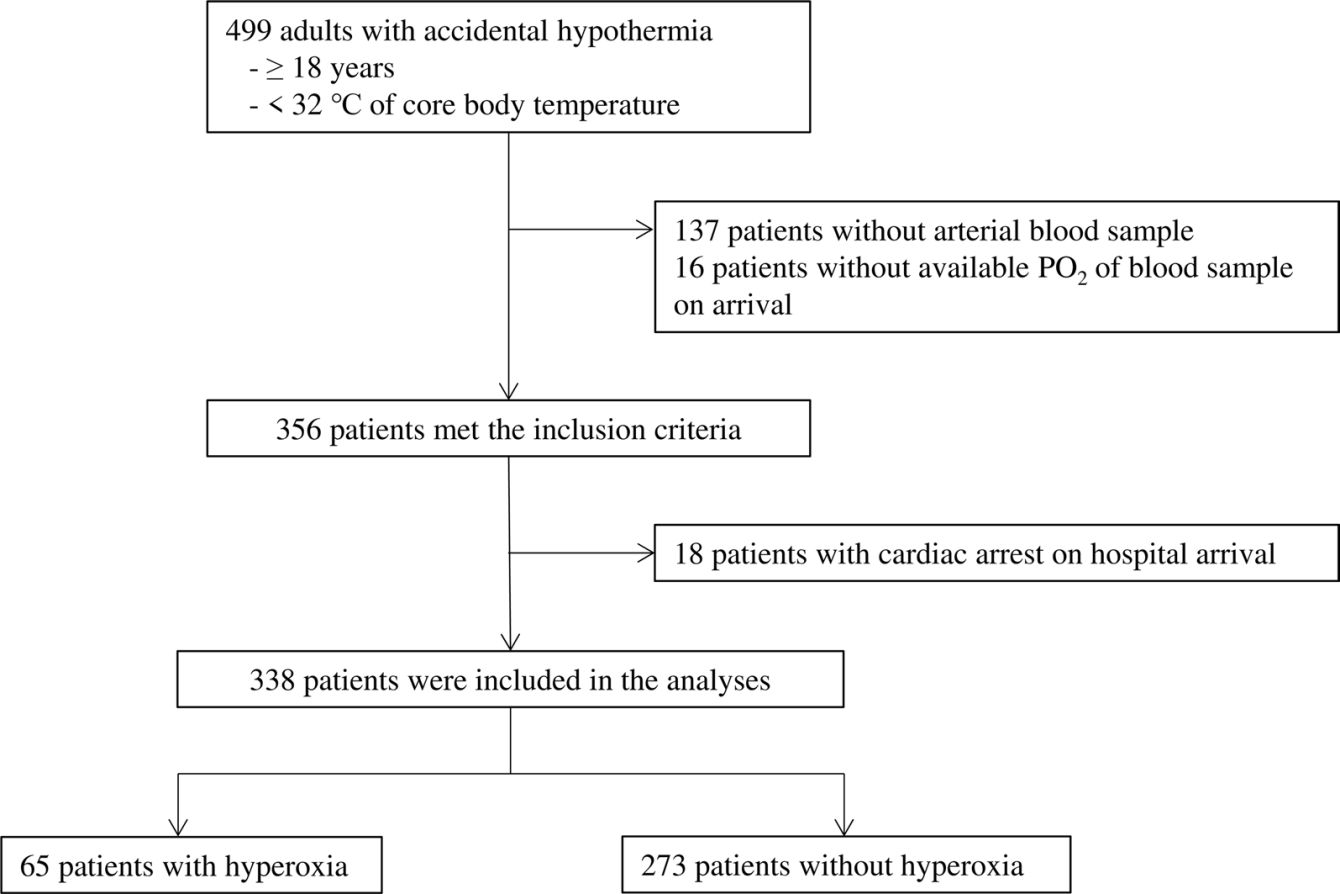
Patient flow diagram. Of the 499 patients with accidental hypothermia, 338 adults had available arterial partial pressure of oxygen (PaO_2_) data at the emergency department and arrived at participating hospitals without cardiac arrest; therefore, they were eligible for this study. In total, 65 patients (19.2%) had hyperoxia with PaO_2_ levels of 300 mmHg or higher before initiating in-hospital rewarming. PO_2_, partial pressure of oxygen

Of the 499 patients with accidental hypothermia in the ICE-CRASH study, 338 adults had available PaO_2_ data at the ED and arrived at participating hospitals without cardiac arrest; therefore, they were eligible for this study (Fig. 1).

In total, 65 patients (19.2%) had hyperoxia with PaO_2_ levels of 300 mmHg or higher before initiating in-hospital rewarming. Patient characteristics are shown in Table 1. The median PaO_2_ level was 366 mmHg in patients with hyperoxia and 135 mmHg in those without. Patients with hyperoxia had higher FiO_2_, Carlson Comorbidity Index, and lactate on arrival, as well as lower GCS, SBP, body temperature, and base excess when compared to those without hyperoxia. Furthermore, a higher proportion of patients with hyperoxia had independent ADL and hypothermia indoors.

**Table 1:** Characteristics of patients with hypothermia

Variables	Before IPW					After IPW				
	Hyperoxia		No hyperoxia		Standardized difference	Hyperoxia		No hyperoxia		Standardized difference
Case	65		273							
PaO_2_, mmHg, median (IQR)	366	(317–431)	135	(90–210)	2.840	361	(315–416)	139	(90–223)	2.779
FiO_2_, median (IQR)	0.9	(0.9–0.9)	0.4	(0.2–0.9)	1.341	0.9	(0.9–0.9)	0.5	(0.2–0.9)	1.089
PF ratio, mmHg, median (IQR)	432	(372–558)	332	(231–545)	0.518	438	(369–552)	318	(221–516)	0.583
Age, years, median (IQR)	83	(72–88)	81	(71–88)	0.069	83	(77–88)	82	(71–88)	0.042
Sex, male, *n* (%)	33	(50.8%)	149	(54.6%)	0.085	147	(50.7%)	152	(51.9%)	0.020
Comorbidity, Charlson index, median (IQR)	1	(0–2)	0	(0–2)	0.207	0	(0–2)	1	(0–2)	0.006
ADL, independent*, *n* (%)	62	(68.1%)	887	(65.2%)	0.166	127	(43.8%)	149	(50.9%)	0.006
Etiology-identified, *n* (%)										
Intoxication	6	(9.2%)	19	(7.0%)	0.083	24	(8.3%)	23	(7.8%)	0.017
Infection	16	(24.6%)	60	(22.0%)	0.062	60	(20.7%)	71	(24.1%)	0.083
Trauma	1	(1.5%)	17	(6.2%)	0.245	8	(2.8%)	8	(2.7%)	0.002
Indoor occurrence, *n* (%)	56	(86.2%)	218	(80.1%)	0.161	249	(85.9%)	247	(84.0%)	0.052
Transportation time, min, median (IQR)	39	(30–57)	41	(33–51)	0.288	40	(30–59)	40	(31–50)	0.038
Vital signs on hospital arrival										
GCS, median (IQR)	7	(6–9)	10	(7–12)	0.519	9	(6–11)	9	(6–11)	0.000
SBP, mmHg, median (IQR)	89	(50–117)	104	(70–134)	0.382	96	(60–125)	93	(61–124)	0.049
HR, /min, median (IQR)	59	(42–78)	64	(48–84)	0.196	64	(45–80)	60	(47–81)	0.021
RR, > 20/min, *n* (%)	16	(24.6%)	71	(26.0%)	0.032	73	(25.3%)	73	(24.9%)	0.008
RR, 10–20/min, *n* (%)	45	(69.2%)	194	(71.1%)	0.040	206	(71.3%)	209	(71.3%)	0.001
RR, < 10/min, *n* (%)	4	(6.2%)	8	(2.9%)	0.155	10	(3.5%)	11	(3.8%)	0.016
BT, °C, median (IQR)	27.9	(26.5–29.6)	29.0	(27.5–30.3)	0.436	28.3	(27.0–30.3)	28.5	(27.0–30.1)	0.023
Laboratory data, median (IQR)										
Lactate, mmol/L	3.0	(1.8–6.3)	2.6	(1.1–6.1)	0.108	2.9	(1.8–5.6)	2.8	(1.3–6.1)	0.027
Base excess, − mmHg	7.5	(13.9– 2.7)	6.4	(11.9–1.4)	0.254	7.5	(2.8–11.9)	6.6	(1.9–13.1)	0.031
Hct, %	36	(30–42)	36	(31–41)	0.036	37	(31–42)	35	(30–41)	0.025
Platelet, 10^3^/μL	160	(108–235)	178	(115–240)	0.135	164	(112–248)	168	(112–232)	0.008
PT-INR	1.2	(1.1–1.5)	1.2	(1.0–1.4)	0.061	1.2	(1.0–1.5)	1.2	(1.0–1.4)	0.002
Creatinine, mg/dL	1.3	(0.9–2.3)	1.2	(0.7–2.2)	0.048	1.4	(0.8–2.3)	1.3	(0.7–2.2)	0.026
Glucose, mg/dL	125	(83–174)	126	(87–186)	0.116	124	(82–171)	130	(92–193)	0.012

*IPW* Inverse probability weighting, *IQR* Interquartile range, *PF ratio* PaO_2_/FiO_2_ ratio, *ADL* Activity of daily living, *GCS* Glasgow Coma Scale, *SBP* Systolic blood pressure, *HR* Heart rate, *RR* Respiratory rate, *BT* Body temperature, *Hct* Hematocrit, and *PT-INR* Prothrombin time-international normalized ratio. *ADL-independent included living independently or with limited help

A propensity model for hyperoxia exposure was developed, and discrimination power was calculated, yielding a *c*-statistic of 0.699 (0.633–0.766). There were no patients excluded from IPW analyses due to missing covariates for propensity score calculation. Table 1 shows the patient characteristics after IPW with standardized differences, where differences in covariates such as patient demographics, comorbidities, hypothermia severity, and vital signs and laboratory data on arrival were successfully attenuated using the propensity score (standardized difference < 0.1). Propensity score distribution is also shown in Additional file 2: Figure S2.

Table 2 summarizes rewarming information after adjusting for patient backgrounds. The use of invasive rewarming devices, including thermoregulated catheters and hemodialysis, the time to rewarming to 33 °C and 36 °C, laboratory data after rewarming, the SOFA score, and mortality on the day of rewarming were comparable between patients with and without hyperoxia. The median PaO_2_ and FiO_2_ levels after rewarming were 90–110 mmHg and 0.2–0.3, respectively, and were comparable between patients with and without hyperoxia.

**Table 2:** Rewarming information in patients with hypothermia

Variables	Hyperoxia		No hyperoxia		Standardized difference
Rewarming with device, *n* (%)					
Intravascular catheter	26	(9.0%)	33	(11.2%)	0.075
Hemodialysis	9	(3.1%)	11	(3.8%)	0.035
Time from arrival to 33 °C, h, median (IQR)	3.7	(2.0–5.0)	3.3	(2.3–4.8)	0.008
Time from 33 to 36 °C, h, median (IQR)	3.6	(3.0–6.0)	3.0	(2.0–6.0)	0.070
Laboratory data after rewarming, median (IQR)					
Lactate, mmol/L	1.5	(1.1–2.8)	1.4	(0.8–2.9)	0.036
Base excess, mmHg	− 2.8	(− 6.4–0.0)	− 2.1	(− 8.4–1.2)	0.077
Oxygenation after rewarming, median (IQR)					
PaO_2_, mmHg	107	(80–120)	91	(71–117)	0.087
FiO_2_	0.2	(0.2–0.3)	0.3	(0.2–0.4)	0.000
SOFA score on admission, median (IQR)	6	(4–9)	7	(4–10)	0.061
Mortality on day of admission, *n* (%)	15	(5.2%)	17	(5.8%)	0.027
Mortality during rewarming, *n* (%)	22	(7.6%)	19	(6.5%)	0.044

*IQR* Interquartile range, *SOFA* Sequential Organ Failure Assessment. Patient backgrounds were adjusted with IPW

### 28-Day mortality and secondary outcomes

Patients with hyperoxia had significantly higher 28-day mortality than those without (25 (39.1%) vs. 51 (19.5%); OR 2.65 (95% CI 1.47–4.78); *p* < 0.001; Table 3), and similar results were obtained in the IPW analyses (34.0% vs. 23.8%; adjusted OR 1.65 (1.14–2.38); *p* = 0.008; Table 3). The three sensitivity analyses also showed a relationship between hyperoxia and increased 28-day mortality (Additional file 3: Table S1).

**Table 3:** Hyperoxia and clinical outcomes

Outcomes	Hyperoxia	No hyperoxia	*p*-value	OR (95% CI)	
28-Day mortality					
Unadjusted, *n*/total (%)	25/64 (39.1%)	51/262 (19.5%)	< 0.001	2.65	(1.47–4.78)
IPW, %	34.0%	23.8%	0.008	1.65	(1.14–2.38)
CPC ≤ 2 at discharge, *n* (%)	56.9%	63.5%		0.76	(0.55–1.06)
Length of treatment, days, mean, median (IQR)					
Hospital-free days to Day 28	6, 0 (0–11)	8, 0 (0–16)	0.021		
ICU-free days to Day 28	16, 23 (0–26)	18, 23 (2–26)	0.012		
Ventilator-free days to Day 28	18, 28 (0–28)	20, 28 (0–28)	0.034		
Adverse events related to hypothermia/rewarming, % (95% CI)					
Ventricular fibrillation	4.5% (2.1–6.9%)	4.1% (1.8–6.4%)		1.10	(0.49–2.45)
Hemorrhage	25.5% (20.5–30.5%)	24.2% (19.3–29.1%)		1.07	(0.74–1.56)
Pneumonia	21.1% (16.4–25.8%)	22.4% (17.7–27.2%)		0.92	(0.62–1.37)
Pancreatitis	6.2% (3.4–9.0%)	2.0% (0.4–3.7%)		3.18	(1.24–8.12)
Acute kidney injury	19.3% (14.8–23.9%)	24.2% (19.3–29.1%)		0.75	(0.50–1.11)

*OR* Odds ratio, *CI* Confidence interval, *IPW* Inverse probability weighting, *CPC* Cerebral performance category, *IQR* Interquartile range, *ICU* Intensive care unit. Secondary outcomes were compared using IPW analyses

**Fig. 2:**
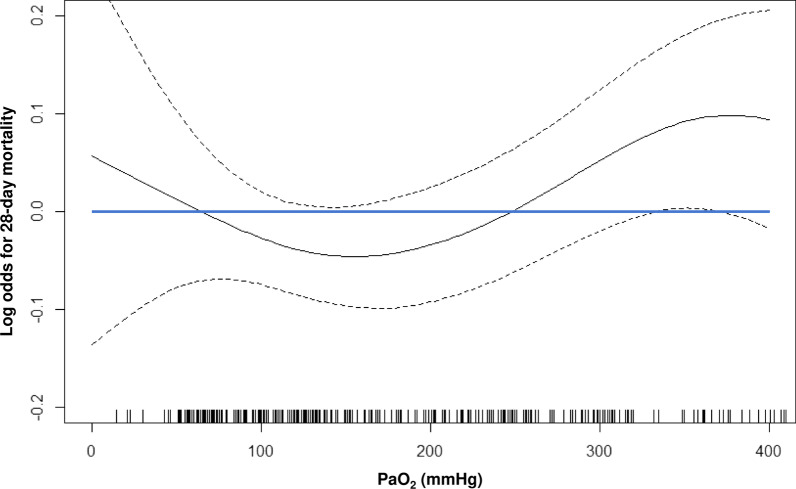
Restricted cubic spline curve of mortality prediction by arterial partial pressure of oxygen. The restricted cubic spline curve of mortality prediction by arterial partial pressure of oxygen (PaO_2_) revealed a convex downward curve of mortality odds as PaO_2_ increased, with PaO_2_ levels of approximately 60–250 mmHg having a lower risk of 28-day mortality among patients with accidental hypothermia

Furthermore, the restricted cubic spline curve of mortality prediction by PaO_2_ revealed a convex downward curve of mortality odds as PaO_2_ increased, with PaO_2_ levels of approximately 60–250 mmHg having a lower risk of 28-day mortality among patients with accidental hypothermia (Fig. 2). In addition, based on the spline curve, 250 mmHg was chosen from the expected upper threshold of low risk of PaO_2_ and 200 mmHg from in the middle of the range of low risk of PaO_2_. Re-defined hyperoxia was associated with increased mortality only with PaO_2_ ≥ 250 mmHg, not with ≥ 200 mmHg (Additional file 3: Table S1).

Moreover, in the post-weight logistic model, both hyperoxia and the trajectory of hyperoxia (average changes in PaO_2_ per hour) were associated with increased 28-day mortality (adjusted OR 3.64 (1.92–6.89) for hyperoxia and 1.02 (1.01–1.03) for 1 mmHg/h decrease in the average change of PaO_2_: slower normalization of PaO_2_ from hyperoxia was associated with higher mortality). Another post-weight logistic model using in-hospital treatments, time duration of rewarming, and the trajectory of hyperoxia similarly revealed that hyperoxia was related to higher 28-day mortality (adjusted OR 2.54 (1.31–4.93)).

The secondary outcomes were summarized in Table 3. Hyperoxia was associated with fewer hospital-, ICU-, and ventilator-free days. In contrast, favorable neurological outcomes at hospital discharge and the frequency of adverse events related to hypothermia or rewarming were comparable between patients with and without hyperoxia, except for pancreatitis, which was more common in patients who experienced hyperoxia before rewarming than in those who did not (6.2% vs. 2.0%; OR 3.18 (1.24–8.12)).

### Subgroup analysis

In subgroup analyses (Table 4), a relationship between increased 28-day mortality and hyperoxia was observed in several subgroups: the elderly at 65 years of age or older, patients with chronic cardiopulmonary diseases, those without hemodynamic instability on hospital arrival, and those with severe hypothermia (OR 1.70 (1.15–2.50), 3.95 (1.45–10.74), 2.73 (1.64–4.56), and 1.69 (1.05–2.73), respectively).

**Table 4:** 28-day mortality in subgroup analyses

Subgroups	Hyperoxia	No hyperoxia	OR	95% CI
*Age*				
< 65 years	10.3% (7.3–19.8%)	11.6% (2.0–21.2%)	0.87	0.22–3.50
≥ 65 years	37.8% (31.7–43.9%)	26.4% (20.8–31.9%)	1.70	1.15–2.50
Chronic cardiopulmonary disease*				
(−)	27.1% (21.6–32.7%)	19.8% (14.8–24.9%)	1.50	0.99–2.30
( +)	78.9% (66.0–91.9%)	48.7% (33.0–64.4%)	3.95	1.45–10.74
*Hemodynamic instability on hospital arrival*				
SBP ≥ 90 mmHg	38.2% (30.9–45.4%)	18.4% (12.3–24.6%)	2.73	1.64–4.56
SBP < 90 mmHg	28.3% (20.0–36.6%)	30.2% (22.3–38.2%)	0.91	0.52–1.59
*Severity of hypothermia*				
< 28 °C	34.9% (27.3–42.4%)	24.0% (17.8–30.3%)	1.69	1.05–2.73
≥ 28 °C	33.1% (25.1–41.1%)	24.3% (16.0–32.6%)	1.54	0.87–2.75
Without hypoxia**	34.0% (28.5–39.5%)	22.2% (17.1–27.3%)	1.81	1.23–2.66

*OR* Odds ratio, *CI* Confidence interval, *SBP* Systolic blood pressure. IPW analyses were performed in each subgroup

*Chronic cardiopulmonary disease included congestive heart failure and chronic lung diseases

**Hypoxia was defined as a PaO_2_ level less than 60 mmHg

In contrast, younger patients (< 65 years), those without chronic cardiopulmonary diseases, those with hemodynamic instability on hospital arrival, and those with non-severe hypothermia had comparable mortality regardless of hyperoxia exposure.

Furthermore, in the subgroup excluding patients with hypoxia (PaO_2_ levels below 60 mmHg), hyperoxia was also associated with higher mortality (OR 1.81 (1.23–2.66)).

## Discussion

This study revealed that the presence of hyperoxia, defined as a PaO_2_ level of 300 mmHg or higher, before the initiation of in-hospital rewarming was associated with an increased 28-day mortality among patients with accidental hypothermia. It remained after adjustment for background characteristics, hypothermia severity, and methods and duration of rewarming. The association between hyperoxia during hospital stay after rewarming and clinical outcomes was not examined in the current study.

One of the pathophysiological mechanisms underlying the harmful effects of hyperoxia in accidental hypothermia is brain tissue injury caused by redundant oxygen [11, 27, 28]. Several studies on traumatic brain injury reported that supranormal oxygen suppressed cell metabolism, resulting in neuronal death [27, 28], and other studies on cerebral reperfusion injury in post–cardiac arrest syndrome showed reduced oxygen delivery due to vasoconstriction caused by hyperoxia [11, 29]. Given that oxygen demand in the brain gradually increases during rewarming from hypothermia [13], continuous exposure to hyperoxia would have paradoxically impeded cerebral oxygenation in patients with accidental hypothermia.

Another possible cause of unfavorable outcomes due to hyperoxia is lung injury caused by supraphysiologic FiO_2_. Previous studies suggested that hyperoxia-induced ALI should be considered when the FiO_2_ level exceeds 0.6–0.7 and becomes problematic when it is greater than 0.8 [30, 31]. In addition, FiO_2_ levels ranging from 0.8 to 1.0 in the first 3 h of resuscitation on severely injured patients were found to increase ICU stay [6]. In the current study, the median FiO_2_ level on arrival was 0.9 in patients with hyperoxia, whereas it was 0.4 in those without hyperoxia. Although FiO_2_ was decreased to 0.2–0.3 after rewarming and the duration of inhaling high oxygen concentrations was unknown, ALI may have developed in patients with hyperoxia. It should also be noted that patients with hyperoxia had longer ventilator usage and ICU stays despite comparable incidence of pneumonia regardless of hyperoxia exposure.

Subgroup analyses suggested that hyperoxia should be avoided particularly in the elderly and patients with chronic cardiopulmonary diseases. Given that the elderly are vulnerable to suboptimal cerebral oxygenation and that those with cardiopulmonary diseases will not tolerate additional lung injuries [32, 33], the adverse effects of hyperoxia would have manifested in such populations. Furthermore, only patients with severe hypothermia (< 28 °C) had unfavorable outcomes following hyperoxia in this study. Because the degree of hypothermia is strongly related to metabolic levels and tissue oxygen requirements [13, 20], unnecessary oxygen would easily accumulate in severe hypothermia, potentially causing clinically obvious harm. However, these results should be interpreted with caution due to the small sample sizes in the subgroups.

Importantly, the findings of this study do not recommend invariably restricted oxygen administration for patients with accidental hypothermia. Although the unfavorable effects of hyperoxia were identified, the restricted spline curve for mortality prediction by PaO_2_ indicated that hypoxia would also cause inappropriate tissue oxygenation. In addition, no adverse effects of hyperoxia were observed in patients with hemodynamic instability, implying that decreased oxygen delivery due to reduced blood flow would have to be compensated for by increasing oxygen content even in patients with accidental hypothermia. Given that there are still unexplained results, such as the increased incidence of pancreatitis in patients with hyperoxia, the pathophysiology of hyperoxia in accidental hypothermia needs to be clarified in future studies.

## Limitations

The findings must be interpreted in the context of the study’s design. We retrospectively retrieved data from the ICE-CRASH study that did not record the indications for administering high FiO_2_ instead of low to moderate FiO_2_. Therefore, our results may differ if the reasons for hyperoxia exposure are dependent on unrecorded, strong prognostic factors, such as the quality of prehospital care and the reliability of peripheral oxygen saturation measurement. However, using the GEE model and adjusting for differences in practice between regions/institutions, the association between hyperoxia and higher mortality was revealed. Another limitation was the lack of clinical information on cerebral and pulmonary functions before, during, and after rewarming. Although supraphysiological oxygen tension would cause brain and lung toxicity, the clinical outcomes of such organ toxicity following hyperoxia cannot be objectively evaluated. Furthermore, the degree and duration of hyperoxia during rewarming were unknown in this study. While hyperoxia before rewarming was identified as a potential harm, the generalizability of the results for oxygen treatment during rewarming is limited. Finally, because hyperoxia was defined as a PaO_2_ level of 300 mmHg or higher based on previous studies, as well as 250 mmHg or higher based on the spline curves, other thresholds for inappropriate PaO_2_ may exist depending on the timing of hyperoxia exposure, hypothermia severity, and characteristics of patients with accidental hypothermia.

## Conclusions

This study revealed that hyperoxia (a PaO_2_ level of 300 mmHg or higher) prior to rewarming was associated with increased 28-day mortality in patients with accidental hypothermia. Restriction of oxygen administration before rewarming should be carefully considered in daily practice, and the appropriate arterial oxygen tension for patients with accidental hypothermia should be validated further in future studies.

## Supplementary Information

**Additional file 1**. **Figure S1**: A Directed Acyclic Graph for the primary analysis model. Relevant covariates for propensity score calculation were selected from known or potential predictors for receiving supraphysiologic amounts of oxygen and predicting clinical outcomes in patients with accidental hypothermia.

**Additional file 2**. **Figure S2**: Propensity score distribution before and after inverse probability weighting. Propensity score distribution was well balanced after inverse probability weighting.

**Additional file 3**.** Table S1**: 28-day mortality in sensitivity analyses.

## Data Availability

The data of this study are available from the ICE-CRASH study group; however, restrictions apply to the availability of these data, which were used under license for the current study and are not publicly available. However, data are available from the authors upon reasonable request and with the permission of the ICE-CRASH study group.

## Abbreviations

FiO_2_: Fraction of inspired oxygen
ALI: Acute lung injury
ICE-CRASH: Intensive Care with Extra Corporeal Membrane Oxygenation Rewarming in Accidentally Severe Hypothermia
ED: Emergency department
ICU: Intensive care unit
PaO_2_: Arterial partial pressure of oxygen
ADL: Activity of daily living
SOFA: Sequential Organ Failure Assessment
CPC: Cerebral performance category
SBP: Systolic blood pressure
IPW: Inverse probability weighting
GEE: Generalized estimating equations
GCS: Glasgow Coma Scale
OR: Odds ratio
IQR: Interquartile range
CI: Confidence interval
