# Supplementary figures and images for: Hyperoxia for accidental hypothermia and increased mortality: a post-hoc analysis of a multicenter prospective observational study
Source: Crit Care. 2023 Apr 1;27:131. doi: 10.1186/s13054-023-04407-8 (PMC10067299; doi:10.1186/s13054-023-04407-8)

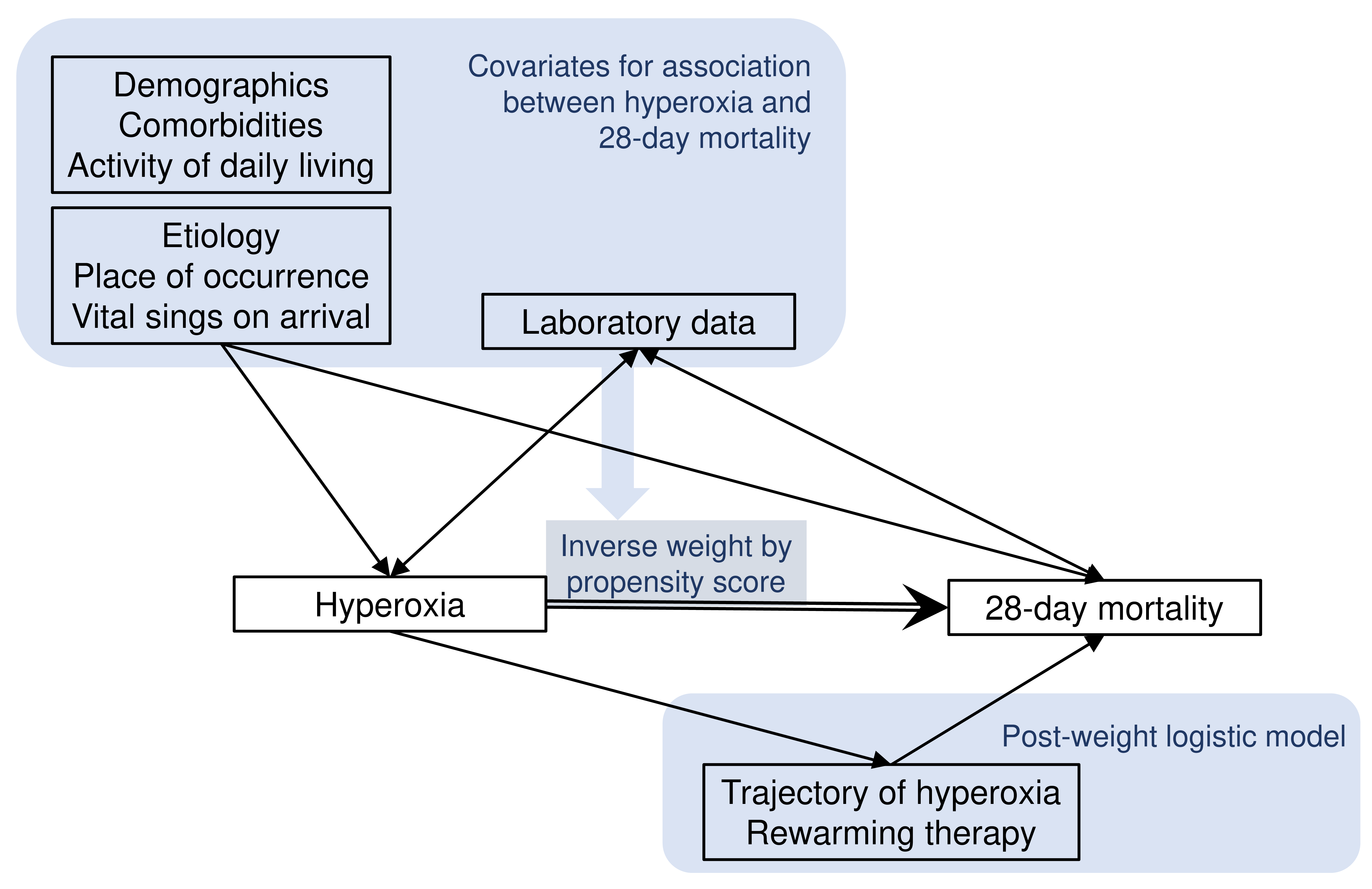

Supplement: Supplementary file 1 — Additional file 1. Figure S1: A Directed Acyclic Graph for the primary analysis model. Relevant covariates for propensity score calculation were selected from known or potential predictors for receiving supraphysiologic amounts of oxygen and predicting clinical outcomes in patients with accidental hypothermia. [file 13054_2023_4407_MOESM1_ESM.tiff]

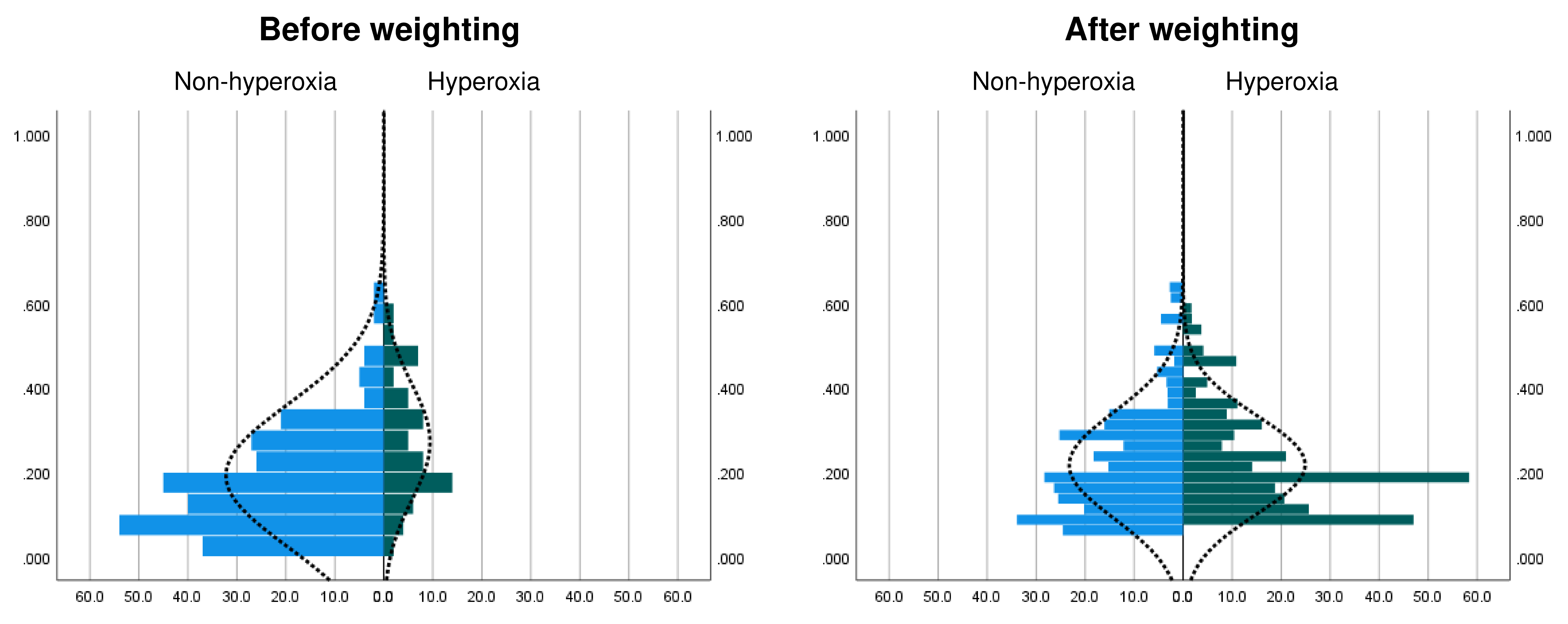

Supplement: Supplementary file 2 — Additional file 2. Figure S2: Propensity score distribution before and after inverse probability weighting. Propensity score distribution was well balanced after inverse probability weighting. [file 13054_2023_4407_MOESM2_ESM.tiff]
