# Supplementary material for: Hyperoxia for accidental hypothermia and increased mortality: a post-hoc analysis of a multicenter prospective observational study
Source: Crit Care. 2023 Apr 1;27:131. doi: 10.1186/s13054-023-04407-8 (PMC10067299; doi:10.1186/s13054-023-04407-8)
Supplement: Supplementary file 3 — Additional file 3. Table S1: 28-day mortality in sensitivity analyses. [file 13054_2023_4407_MOESM3_ESM.docx]

| eTable S1. 28-day mortality in sensitivity analyses | | | | | |  |
| --- | --- | --- | --- | --- | --- | --- |
| 28-day mortality, *%* (*95% CI*) | | Hyperoxia | No hyperoxia | OR | 95% CI |  |
|  | Generalized estimating equations |  |  | 2.05 | 1.02–4.09 |  |
|  | Multivariate logistic regression* |  |  | 2.18 | 1.11–4.30 |  |
|  | IPW without restriction** | 38.8% (33.3%–44.2%) | 21.5% (16.9%–26.0%) | 2.32 | 1.63–3.30 |  |
|  | Hyperoxia defined as PaO2 levels of 250 mmHg or higher*** |  |  | 1.56 | 1.09–2.23 |  |
|  | Hyperoxia defined as PaO2 levels of 200 mmHg or higher*** |  |  | 1.35 | 0.94–1.96 |  |
| OR, odds ratio; CI, confidence interval; IPW, inverse probability weighting; GEE, generalized estimating equation. *Final model included hyperoxia, age, activity of daily living, Glasgow Coma Scale, hematocrit, prothrombin time, and creatinine, as validated mortality predictors. **All patients were included regardless of propensity score. ***IPW analyses were repeated. | | | | | |  |
|  |  |  |  |  |  |  |
|  |  |  |  |  |  |  |
|  |  |  |  |  |  |  |
|  |  |  |  |  |  |  |
